# Development of a Novel Anti-Obesity Compound with Inhibiting Properties on the Lipid Accumulation in 3T3-L1 Adipocytes

**DOI:** 10.29252/ibj.24.3.155

**Published:** 2019-11-30

**Authors:** Moloud Payab, Shirin Hasani-Ranjbar, Maryam Baeeri, Mahban Rahimifard, Babak Arjmand, Hamed Haghi-Aminjan, Mohammad Abdollahi, Bagher Larijani

**Affiliations:** 1 Obesity and Eating Habits Research Center, Endocrinology and Metabolism Molecular-Cellular Sciences Institute, Tehran University of Medical Sciences, Tehran, Iran;; 2 Endocrinology and Metabolism Research Center, Endocrinology and Metabolism Clinical Sciences Institute, Tehran University of Medical Sciences, Tehran, Iran;; 3 Toxicology and Diseases Group, Pharmaceutical Sciences Research Center, Tehran University of Medical Sciences, Tehran, Iran;; 4 Cell Therapy and Regenerative Medicine Research Center, Endocrinology and Metabolism Molecular-Cellular Sciences Institute, Tehran University of Medical Sciences, Tehran, Iran;; 5 Metabolomics and Genomics Research Center, Endocrinology and Metabolism Molecular-Cellular Sciences Institute, Tehran University of Medical Sciences, Tehran, Iran;; 6 Pharmaceutical Sciences Research Center, Ardabil University of Medical Sciences, Ardabil, Iran;; 7 Department of Toxicology and Pharmacology, Faculty of Pharmacy, Tehran University of Medical Sciences, Tehran, Iran

**Keywords:** Berberine, Capsaicin, Catechin, Obesity, 3T3-L1 cells

## Abstract

**Background:**

Obesity as a developing global challenge can be characterized by increase in adipocyte number and size arising from adipogenesis. Control of adipogenesis, as a potential strategy, can prevent and manage obesity. So far, the effectiveness of herbal medicine and active ingredients therapies for obesity and metabolic syndrome treatment has been investigated. In this study, a novel combination of berberine, catechin, and capsaicin was developed, and their effect on 3T3-L1 adipocytes was investigated.

**Methods:**

The effect of active ingredient on the cell viability was assessed by MTT assay. Adipocytes were treated with various concentrations of berberine (3 and 6.25 μM), catechin (6.25 and 12.5 μM), and capsaicin (6.25 and 12.5 μM) alone and in combination.

**Results:**

All active ingredients did not affect the cell viability by MTT assay at different concentrations. The dual and triple combinations of three active ingredients showed excellent potential as anti-obese without any toxicity. The inhibitory effect of berberine, catechin, and capsaicin on the differentiation of 3T3-L1 preadipocytes was found to be dose-dependent. These results indicate that catechin in both doses may have a stronger effect than the two other active ingredients on the intracellular lipid accumulation. Also, the triple combination of the aforementioned ingredients showed better responses than their dual combination.

**Conclusion:**

This work is the first report to simultaneously investigate these three active ingredients in a single, dual, and triple formats. The berberine, catechin, and capsaicin co-treatment inhibits the adipogenesis during the differentiation process. This compound can be a prospective therapy for obesity and relevant diseases such as dyslipidemia.

## INTRODUCTION

Obesity is described as an extreme body fat accumulation, which is resulted from the imbalance between energy input and output^[^^1^^]^, and has a significant contribution to several chronic diseases, especially type 2 diabetes mellitus, cardiovascular diseases, sleep disorders, dyslipidemia, osteoarthritis, and some sorts of cancers^[^^2^^-^^4^^]^. Obesity can be characterized by an increment in adipocyte number and size resulting from adipogenesis (the differentiation of preadipocytes toward mature adipocytes). Various contributing factors are found to have an influential impact on the hyperplasia (number) and hypertrophy (size) of adipocytes^[^^5^^]^. According to the World Health Organization, in 2016, over 1.9 billion (39%) individuals aged 18 years and older were overweight, among which more than 650 million (13%) were considered as obese^[^^6^^]^. Obesity is mainly due to the accumulation of TG in adipocytes and an increase in their size. Control of adipogenesis as a possible approach can be useful to prevent and treat obesity, and the adipocyte differentiation is a major player in the adipose tissue growth^[^^7^^]^.

So far, the effectiveness of hundreds of herbs and active ingredients has been investigated for the obesity and metabolic syndrome treatment. For instance, the active ingredient of berberine, found in the plants of *Rhizoma coptidis* and *Cortex phellodendri*, has traditionally been applied for the treatment of infection and diabetes^[^^8^^]^. Some studies, have indicated that berberine, an isoquinoline alkaloid, can improve the metabolic syndrome and reduce plasma TG and cholesterol levels^[^^9^^, ^^10^^]^. Investigations on the impacts of berberine on obesity have revealed the adipogenesis inhibitory effects of this compound during the differentiating of 3T3-L1 preadipocytes^[^^7^^,^^8^^,^^11^^,^^12^^]^. A recent study has shown that this active ingredient can reduce the amount of TG accumulation through enhancing the expression of adipose TG lipase (*Atgl*) enzyme in 3T3-L1 adipocytes^[^^13^^]^. Berberine also increases the expression of brown adipose tissue marker and uncoupling protein 1, as well as promoting the differentiation of white into brown adipose tissues. Accordingly, berberine can strongly be helpful for thermogenesis and body weight loss^[^^10^^]^.

Animal studies have demonstrated that the oral administration of green tea (*Camellia sinensis*) reduces the adipose tissue weight, and active ingredients in green tea, like catechin, may suppress adipocyte differentiation^[^^14^^]^. Another active ingredient for the treatment of obesity is capsaicin in placental tissue of red pepper. The consumption of this ingredient, which possesses an anti-adiposity property, has been associated with energy expenditure and reduced food intake^[^^15^^,^^16^^]^.

The combination of some effective herbs and their active ingredients can increase their therapeutic effect. As an example, the compound Huanghousu synthesized from magnolol and berberine has been reported to improve lipid metabolism better than a single component^[^^17^^]^. Also, supplementation with catechin and resveratrol could enhance fat oxidation^[^^18^^]^.

In this study, the design and effectiveness of a unique and an effectual composition, including berberine, catechin, and capsaicin, with potential use in treatment of obesity were studied. 

## MATERIALS AND METHODS

**Materials and chemical** **s**

The 3T3-L1 cells were procured from the Iranian Biological Resource Center, Tehran, Iran. Berberine, capsaicin, and catechin (purity > 98%) were obtained from the Santa Cruz Biotechnology, USA. PBS, DMEM, and FBS were all purchased from Sigma-Aldrich Company (GmbH Munich, Germany). INS, IBMX, and Oil Red O were acquired from Sigma Aldrich, USA, DEXA from Santa Cruz Biotechnology (CA, USA), ethanol and n-butanol from Merck Chemical Co. (Germany), and MTT from Sigma-Aldrich (Germany).

**Viability of 3T3-L1 cells**

The effect of active ingredients on cell viability was assessed by MTT assay. The 3T3-L1 cells were seeded onto 96-well plates at 1 × 10^4^ cells/well and maintained overnight. Cells were treated with different concentrations of berberine^[^^8^^]^, capsaicin^[^^19^^,^^20^^]^ (3, 6.25, 12.5, 25, 50, and 100 μM) and catechin (6.25 , 12.5, 25, 50, 100, and 200 μM)^[^^21^^]^ along with the control for 24 hours. Subsequently, 50 μl of 5 mg/ml solution of MTT was added to each sample and incubated for 3 hours (5% CO_2_ and 37 °C). In order to dissolve MTT formazan, DMSO was added. The absorbance was measured at 570 and 690 nm by ELISA microplate reader (Synergy, BioTek Instruments Inc., Germany). 

**Cell culture and differentiation**

The 3T3-L1 preadipocytes were cultured in DMEM (10% FBS, 100 units/mL of penicillin, and 100 μg/mL of streptomycin in a humidified 5% CO_2_ incubator at 37 °C for 10-12 days), while refreshing every 2 days.

**Fig. 1:**
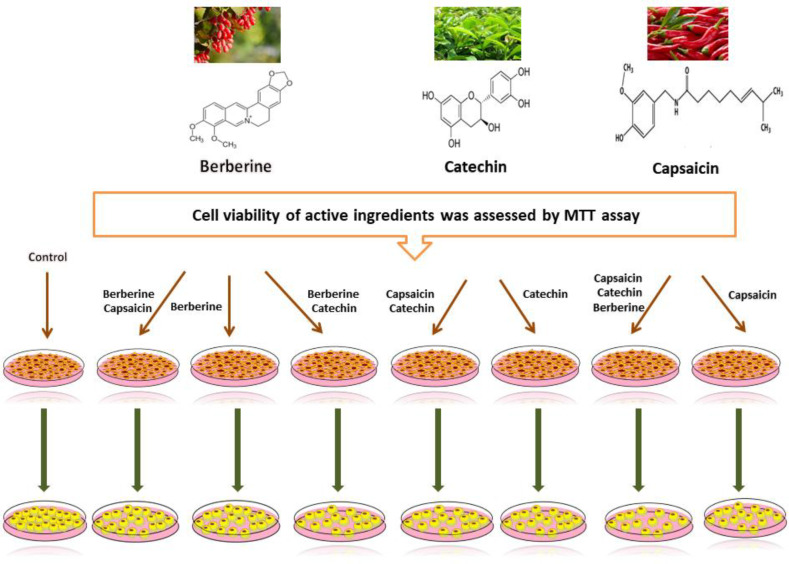
The methodology of the experimental study

The 3T3-L1 preadipocytes were seeded on a six-well plate at a density of 2 × 10^5^ cells/well. Cells were grown in DMEM until a confluency of 70% for the induction of adipose differentiation (day 0). For differentiation into mature adipocytes, cells were fed with a differentiation medium (by adding 0.25 μM of DEXA, 0.5 mM of IBMX, and 5 μg/mL of INS in DMEM containing 10% FBS) for two days (day 3). After that, the medium was refreshed, and the cells were stored in DMEM containing 10% FBS and 5 μg/mL of INS, and the medium was refreshed on a two-day basis. On day 12, the differentiation of 3T3-L1 into mature adipocytes occurred with an approximate rate of 90%^[^^22^^]^. During this time period, the 3T3-L1 cells were treated with active ingredients (berberine, catechin, and capsaicin alone or their combination) with different concentrations, and the effect of these treatments on lipid cumulation by adipocytes was examined by Oil Red O staining method (Fig. 1). Cells were treated with different concentrations of berberine (3 and 6.25 μM), catechin (6.25, and 12.5 μM), and capsaicin (6.25 and 12.5 μM). The negative and positive controls, in the present study, were preadipocytes treated with the similar amounts of DMSO. 

**Measuring lipid accumulation by Oil Red O staining**

The differentiated adipocytes were rinsed twice with PBS, followed by 10% buffered formalin as a fixator at room temperature for 1 hour. After removing this solution, the cells were washed by distilled water and stained in a freshly diluted Oil Red O solution (0.3% Oil Red O solution, 60% isopropanol, and 40% water; Sigma-Aldrich, USA) at room temperature for 1 hour. Then the cells were washed twice with distilled water and counterstained with hematoxylin for 10 seconds^[^^23^^]^. The cells were photographed and the lipid droplets were observed and counted by using an inverted microscope (TS100, Nikon, Tokyo, Japan). To quantify lipid accumulation, the Oil Red O stained-cells were washed twice with 60% isopropanol and destained with isopropanol at 25 °C for 10 minutes. The absorbance of the extracted Oil Red O dye from the stained cells was then read at 540 nm by a microplate reader (Synergy, BioTek Instruments Inc., Germany).

**Statistical analysis**

Six repetitions of independent experiments were conducted. In the current study, the mean ± SEM was used to present the data. Also, one-way analysis of variance (ANOVA) and Tukey’s multi-comparison tests were conducted using StatsDirect (version 3.2.8), in order to define the statistical difference (*p* < 0.05) across the treated and control groups. 

**Ethical statement**

The study was approved by the Ethics Committee of Endocrinology Metabolism Research Institute, Tehran University of Medical Sciences, Tehran, Iran (approval code: IR.TUMS.EMRI.REC.1395.0091).

## RESULTS

**Effects of berberine, catechin, and capsaicin on 3T3-L1 preadipocyte cell viability**

**Fig. 2:**
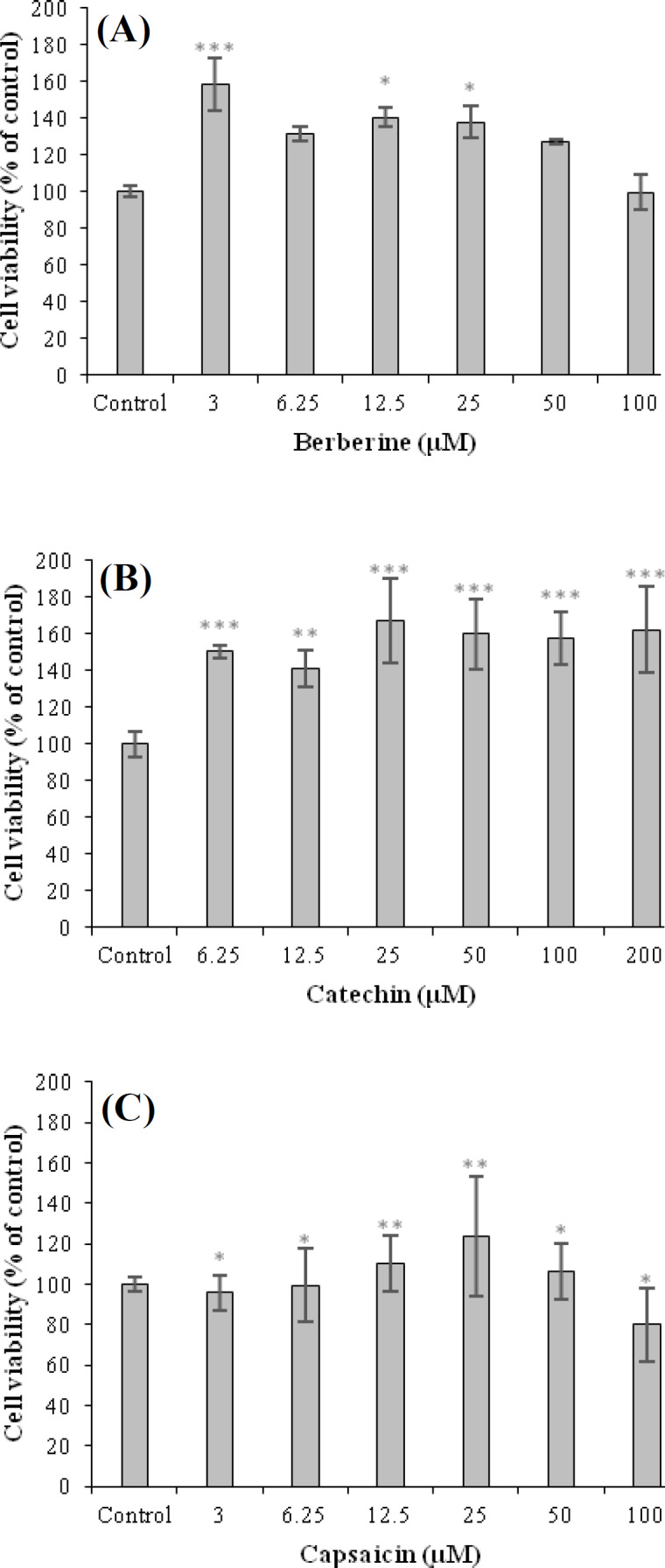
The effects of berberine (A), catechin (B), and capsaicin (C) on the viability of 3T3-L1 cells. The cell viability of active ingredients was assessed by MTT assay. Cells were treated with various concentrations of berberine and capsaicin (3, 6.25, 12.5, 25, 50, and 100 µM), catechin (6.25, 12.5, 25, 50, 100 and 200 µM) and control group for 24 hours. The absorbance was read at 570 nm by an ELISA microplate reader. Results are presented as the mean ± SEM (n = 6). ^*^p ≤ 0.05, ^**^p ≤ 0.01, and ^***^p ≤ 0.001 are considered as a significant difference from the control group

In order to assess the effect of these compounds on viability of 3T3-L1 cells, the MTT assay was carried out. Accordingly, cells were treated with the various concentrations of berberine, capsaicin (3, 6.25, 12.5, 25, 50, and 100 µM) and catechin (6.25, 12.5, 25, 50, 100, and 200 µM). As shown in Figure 2, all active ingredients did not affect cell viability at different concentrations. The results indicated that berberine and capsaicin did not alter the cell viability at concentrations in the range of 3–100 µM (Fig. 2A and 2C). Also, catechin had no effect on the cell viability at concentrations between 6.25 and 200 µM (Fig. 2B). Thus, we used the three compounds at various concentrations for subsequent experiments. Based on our findings, the non-cytotoxic concentrations of the mentioned compounds were selected to follow the inhibitory impacts of these compounds on lipid cumulating.

**Effect of berberine, catechin, and capsaicin on the lipid accumulation in 3T3-L1 adipocytes**

**Fig. 3:**
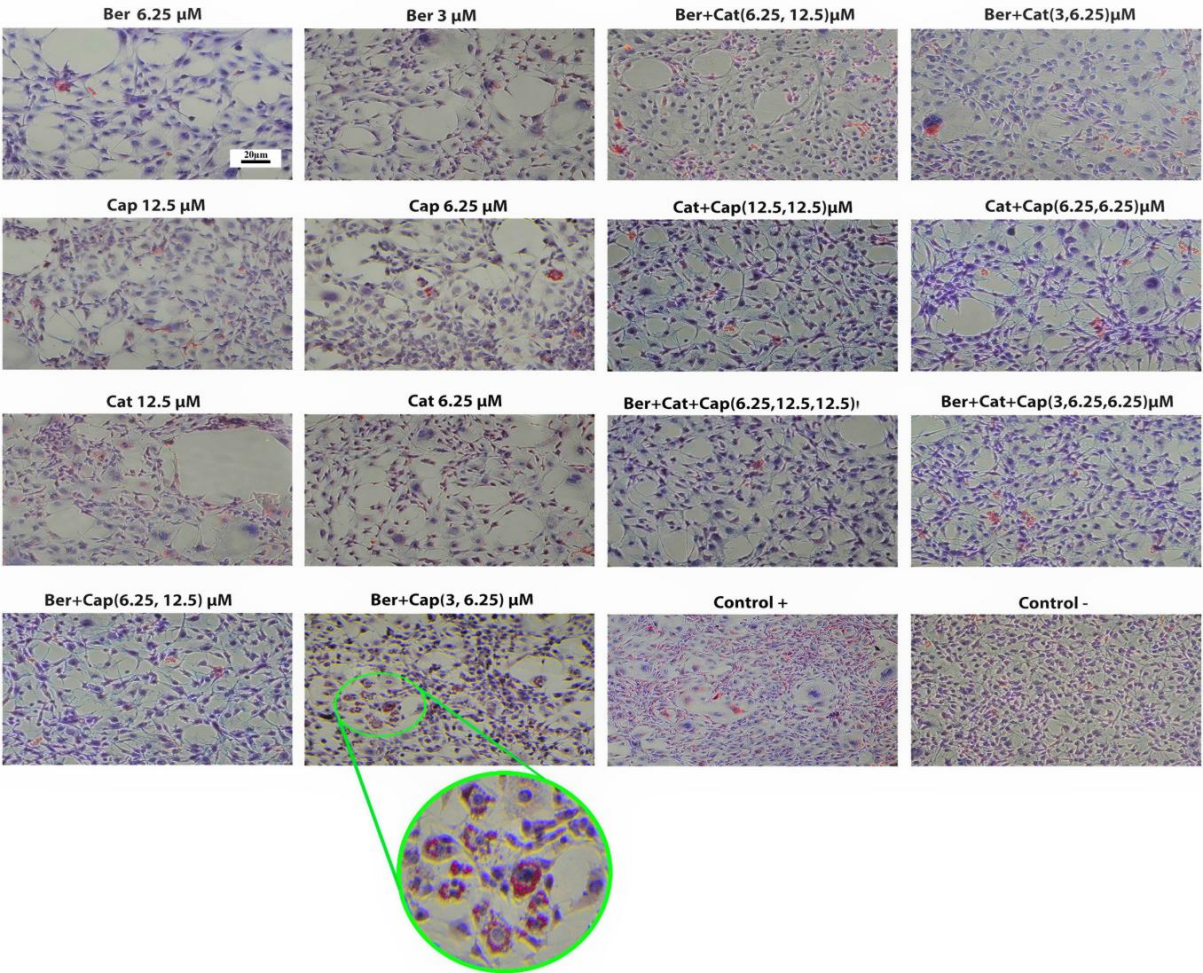
Reduction in the lipid droplet accumulation in 3T3-L1 cells by berberine, catechin, and capsaicin co-treatment. Representative images were randomly selected, and sections of 3T3-L1 adipocytes were stained with Oil Red O treated with various concentrations of active ingredients alone and their combination. Scale bars = 20

The 3T3-L1 preadipocytes were differentiated into mature adipocytes by 10 to 12 days. The anti-obesity potential of the three compounds was studied by determining pre-adipocyte differentiation into adipocytes. Oil Red O staining was applied to investigate the intracellular lipid cumulating in 3T3-L1 preadipocytes. The inhibitory effects of berberine, catechin, and capsaicin on preadipocytes differentiation were dose-dependent. Also, for the combination phases, the concentrations of catechin (6.25 μM), capsaicin (12.5 μM), and berberine (3 μM and 6.25 μM) were used. In microscopic examinations using Oil Red O staining, the number and the size of lipid droplets in adipocytes were found to be considerablyless than the control group after treatment with the active ingredients (Fig. 3). Also, the quantitative measurements revealed that the intervention groups had less intracellular lipids accumulation. In the quantitative measurements, after washing the Oil Red O stain, the results indicated that the intracellular accumulation of lipids in 3T3-L1 preadipocytes was notably lower than other groups and the group of control after treatment with catechin (Table 1). 

As shown in Table 1, among the active ingredients studied, catechin showed the highest efficacy in both doses of 6.25 and 12.5 μM; however, catechin caused the strongest inhibition of lipid accumulation among the compounds tested. The 12.5-μM catechin showed a remarkable reduction in the intracellular lipid accumulation in comparison with the other groups. The triple combination of berberine, capsaicin, and catechin indicated better responses than their dual combination, and the combination of 12.5 μM each of capsaicin and catechin was effective in a dual format. These results of our study indicate that triple combination may have a stronger effect than dual ones in case of inhibiting the adipocyte differentiation.

## DISCUSSION

The adipogenesis and accumulation of lipids in adipocytes lead to obesity. Hence, reducing the amount of lipids stored in adipocytes is momentous for obesity preventing and treating. Several natural plant extracts and their active ingredients are found to be influential in inhibiting adipogenesis^[^^24^^]^. Some of the latest investigations have indicated the anti-adipogenic roles of natural plants, including *Coptidis rhizoma* (berberine), green tea (catechin), and red pepper (capsaicin), which have anti-obesity properties^[^^8^^,^^15^^,^^21^^]^. The purpose of the present study was to assess the anti-adipogenic properties of berberine, catechin, capsaicin during single or multiple treatments. Our findings indicated that these compounds alone or in dual and triple combinations decreased the lipid content in adipocytes, and that these active ingredients may have possible application as an anti-obesity impact. The results of the cell viability assays indicated that the reduced accumulation of lipid in 3T3-L1 adipocytes treated with the active ingredients was not due to their toxicity but because of antilipogenic properties.

**Table 1:** Effects of active ingredients (berberine, catechin, and capsaicin alone and their combination) with different concentrations on the intracellular lipids accumulation in 3T3-L1 cells after Oil Red O staining

**Components**	**Dose (μM)**	**Mean (SD)**	***p*** ** value**
Berberine	3.00	107.89 ± 4.49	NS
	6.25	121.58 ± 6.1	**
			
Capsaicin	6.25	103.16 ± 5.15	**
	12.5	113.68 ± 5.48	***
			
Catechin	6.25	94.21 ± 4.51	***
	12.5	80.00 ± 4.2	***
			
Berberine + capsaicin	3.00 + 6.25	157.89 ± 7.50	***
	6.25 + 12.5	103.16 ± 5.35	NS
Berberine + catechin	3.00 + 6.25	131.05 ± 6.2	*
	6.25 + 12.5	117.89 ± 6.1	NS
			
Capsaicin + catechin	6.25 + 6.25	127.89 ± 6.39	*
	12.5 + 12.5	114.74 ± 5.79	NS
			
Berberine + capsaicin + catechin	3.00 + 6.25 + 6.25	116.55 ± 5.62	*
	6.25 + 12.5 + 12.5	112.84 ± 5.14	*
			
Con^+^	-	147.37 ± 7.36	
			
Con^-^	**-**	** 100 ± 4.70**	***

The negative (Con^-^) and positive (Con^+^) controls were preadipocytes treated with 0.1% DMSO. Results are presented as the mean ± SD from three independent experiments (^*^p < 0.05, ^**^p < 0.01, ^***^p < 0.001 compared with the control group). NS, not significant

*In vitro* and *in vivo* investigations have shown that the intracellular lipid accumulation and adipocyte size decrease after treatment with berberine in a dose-dependent manner^[^^8^^,^^10^^,^^25^^]^. Moreover, our research has confirmed that berberine in doses of 3 μM and 6.25 μM reduced the lipid accumulation both in size and in number. Studies have pinpointed that berberine can inhibit adipocyte differentiation through various mechanisms^[^^26^^,^^27^^]^. Berberine plays a role in the up-regulation of genes involved in energy expenditure and down-regulation of genes involved in adipogenesis^[^^12^^]^. Berberine decreases the expressions of peroxisome PPARγ, C/EBPα, where they act as key transcription factors at the initial steps of differentiation as well as other adipogenic genes like SREBP-1c, Scd1, FASN, and ACC^[^^13^^,^^26^^,^^28^^]^. Also, berberine up-regulates the expression of *Atg1*^[^^13^^]^. 

Another effective active ingredient in the adipogenesis inhibition, investigated in the present research, was capsaicin, which was used at the doses of 6.25 μM and 12.5 μM. Previous studies have revealed that capsaicin inhibits the adipocyte differentiation and intracellular lipid accumulation^[^^29^^,^^30^^]^. Findings from a study revealed that capsaicin plays a dual role in inhibiting adipogenesis, activating transient receptor potential cation channel subfamily V member 1, and inducing brown-like phenotype at higher doses in 3T3-L1 adipocytes. The anti-adipogenic effects of capsaicin are associated with brown-like phenotype^[^^31^^]^. Additionally, capsaicin decreases the *PPARγ, C/EBPα*, and leptin expression and increases the lipoprotein lipase expression, indicating the ability of capsaicin to inhibit the 3T3-L1 preadipocytes differentiation^[^^29^^]^.

Besides energy efficiency and feeding control, some other strategies, including increasing energy expenditure, controlling fat, and carbohydrate absorption, as well as proliferation and differentiation inhibiting of preadipocytes have been introduced for the obesity treatment^[^^32^^]^. In the present study, doses of 6.25 μM and 12.5 μM were used for catechin, which has many inhibitory effects on obesity. Catechin decreases *PPARγ* and *C/EBPα* (key transcription factors of the initial stage of transcription) expression, thus inhibiting the differentiation of fat cells^[^^21^^]^.

*PGC-1α* has a key function in the carbohydrate, lipid, and energy homeostasis. As an energy regulator, *PGC-1α* accelerates the oxidation of the fatty acids by enhancing the function and activity of mitochondria, which has an effect on the body's energy expenditure^[^^33^^,^^34^^]^. The overexpression of *PGC-1α* gene is performed by catechin through the promoter activation^[^^35^^]^.Some studies have also pointed out that catechin has an impact on fatty acids oxidation and energy expenditure by the genes expression regulation in adipose tissue, including the expression of hormone-sensitive lipase and *UCP1* gene^[^^36^^]^.

So far, no study has been conducted to examine the simultaneous effects of these three active components for the inhibition of adipogenesis. In one study, the inhibitory effects of capsaicin and catechin on adipocytes differentiation was investigated and these two compound showed to inhibit adipogenic activity via promoting Adenosine monophosphate-activated kinase, one of the primary targets for the inhibition of adipocyte differentiation and controlling adipogenesis^[^^30^^]^.

According to the results of the present study, the dual and triple combinations of berberine, catechin, and capsaicin have excellent potential as effective anti-obesity agents without any toxicity. The co-treatment of the mentioned compounds inhibited the adipogenesis during the differentiation process. This compound can be a potential therapy for obesity and relevant diseases such as dyslipidemia. Considering the effectiveness of the aforementioned active ingredients, the present study paves the way for future animal and human studies. More researches are required to explore the molecular mechanisms of these active ingredients. One of the strengths of this study is that the active ingredients (berberine, catechin, and capsaicin) that examined by various mechanisms have the ability to inhibit the intracellular lipid accumulation.
